# RSCNN-PseU: random searching-based convolutional neural network model for identifying RNA pseudouridine

**DOI:** 10.1093/bib/bbaf417

**Published:** 2025-08-15

**Authors:** Jian Jin, Jie Feng

**Affiliations:** School of Science, Minzu University of China, No. 27 Zhongguancun South Street, Haidian District, Beijing 100081, China; School of Science, Minzu University of China, No. 27 Zhongguancun South Street, Haidian District, Beijing 100081, China

**Keywords:** RNA pseudouridine, discrete Fourier transform, convolutional neural network, random searching

## Abstract

In order to identify RNA pseudouridine more effectively, in this paper, we propose a new feature extraction method. First, the original sequence is converted into a numerical sequence based on two physicochemical properties of dinucleotides, namely free energy and hydrophilicity; then, it is subjected to discrete Fourier transform (DFT) and the amplitude of each DFT value is calculated. In this way, for an RNA sequence of length *N*, we can obtain 2(*N*-1) features. Ultimately, we utilize a convolutional neural network for prediction, incorporating a dynamic fully connected layer within it. The random search algorithm is employed to ascertain the optimal number of fully connected layers and to fine-tune the model parameters, thereby enabling adaptive regulation of model complexity and accommodating the varying needs of different species and datasets. Experimental results have shown that our model RSCNN-PseU has better identification effect for RNA pseudouridine.

## Introduction

Pseudouridine is the most abundant modified nucleoside on RNA, also known as the “fifth nucleoside” of RNA [1]. As the 5-position isomer of uridine, there are two main mechanisms for the formation of pseudouridine in eukaryotes: dependence on pseudouridine synthase or dependence on the H/ACA ribonucleoprotein complex. This modified nucleoside is widely found in multiple types of RNAs from several species, including tRNAs, mRNAs, snRNAs, and snoRNAs.

Pseudouridine plays an important role in RNA. First, it can base pair with adenosine like uridine, but pseudouridine alters RNA structure by improving base pairing, base stacking, and backbone stability. Second, pseudouridine has the biological functions of reducing mRNA immunogenicity, improving the stability of mRNAs, and enhancing their translation ability. Therefore, in mRNA vaccine and drug development, researchers incorporate pseudouridine or its analogs to replace uridine into mRNA to solve the problem that mRNA drugs are easily recognized by the immune system and cleared, resulting in immune side reactions [2].

Many methods have been proposed for the identification of pseudouridine. In the early days, researchers mainly relied on traditional chemical experimental methods, which could identify short sequences [3, 4] and were therefore not suitable for large-scale identification. Subsequently, with the development of high-throughput sequencing technology, some high-throughput sequencing methods targeting pseudouridine have emerged, such as Pseudo-seq [5], Ψ-seq [6], PSI-seq [7], etc. These methods can identify pseudouridine in RNA in large quantities, but they all require sequencing instruments and are very expensive in terms of materials, resulting in high costs.

With the rapid development of big data technology, predictive models based on machine learning [8–16] and deep learning [17–20] have been widely used in pseudouridine identification. In Machine Learning approaches, researchers achieved significant accuracy (ACC) improvements through multimodal feature fusion and optimization strategies. For example, Wang *et al.* [8] fused six individual predictor variables into one integrated predictor variable through a parallel fusion strategy and finally made a prediction through an integrated learning method. He *et al.* [9] obtained an effective feature subset by combining five different features; then, a sequential forward feature selection strategy was used, and the model was constructed using a support vector machine (SVM) based on the selected feature subset. Chen *et al.* [13] proposed a twin support vector machine (TWSVM)-based fake uridine recognition model which combines several feature representation techniques and uses maximum correlation and minimum redundancy methods to obtain the optimal subset of features for training. Chen *et al.* [15] proposed the PseU-KeMRF predictor, which employs a multimodal feature encoding strategy integrating four RNA sequence representations: binary features, single-strand position-specific trinucleotide propensity, nucleotide chemical properties, and pseudo k-tuple composition. The model utilizes SVM recursive feature elimination for feature subset optimization, followed by a polynomial kernel-enhanced random forest classifier for cross-validation and independent testing. Meanwhile, Wang *et al.* [16] developed the PseUpred-ELPSO framework, which innovatively constructs a 30D RNA dynamic map through nucleotide composition bias analysis. This approach first generates sequence representations using five tree-based classifiers (including XGBoost and LightGBM) as base learners, subsequently applies particle swarm optimization (PSO) for adaptive weight adjustment of the feature map, and ultimately achieves enhanced prediction robustness through a stacked logistic regression meta-classifier for decision fusion.

In Deep Learning developments, architectural innovations enhanced feature abstraction. Khan *et al.* [17] input the original RNA sequences and the predicted secondary structures into two sets of convolutional neural networks (CNNs) for prediction. Zhuang *et al.* [18] proposed a deep learning framework, called PseUdeep. In this method, three encoding methods are used to extract the features of RNA sequences, i.e. one-hot encoding, K-mer nucleotide frequency pattern, and position-specific nucleotide composition. The three feature matrices are convoluted twice and fed into the capsule neural network and bidirectional gated recurrent unit network with a self-attention mechanism for classification. Recent advances in deep learning, such as transformer-based models [20], have shown promise in RNA modification prediction. However, their high computational costs limit applications compared to CNNs.

**Figure 1:**
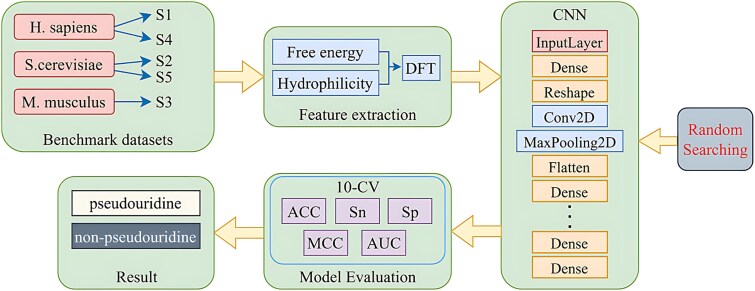
Flowchart of the RSCNN-PseU modeling framework.

Based on the previous studies, in this paper, we propose a new model for the identification of pseudouridine, called RSCNN-PseU. The flowchart of RSCNN-PseU is shown in Fig. 1. First, we convert the original sequence into a digital sequence based on the two physicochemical properties of dinucleotides, which are free energy and hydrophilicity. Next, we perform a discrete Fourier transform (DFT) on it and calculate the amplitude of each DFT value. Then, a CNN was utilized for prediction, which was accessed in the model with dynamic fully connected layers, and the random search algorithm was used to determine the number of layers in the fully connected layers, and the relevant parameters in the model were optimized. Finally, experiments are conducted on five datasets and the models are evaluated using a 10-fold cross-validation method. The results not only show that the new feature extraction model is effective for identifying RNA pseudouridine but also demonstrate the superiority of CNN-PseU for RNA pseudouridine identification.

## Materials and methods

### Benchmark datasets

In this paper, we use the datasets created by Chen *et al.* [21]. They first used the CD-HIT software to remove redundancy and then used a randomly picking procedure on the negative samples that did not contain pseudouridine so that there were as many positive as negative samples. The sequences were then aligned so that the uridine was in the center of the sequence, and the sequences of the same species were of the same length through an interception operation.

Three training datasets were finalized: human (*Homo sapiens*, *H. sapiens*), yeast (*Saccharomyces cerevisiae*, *S. cerevisiae*), and mouse (*Mus musculus*, *M. musculus*). In addition to the training datasets, independent datasets were also added for humans and yeast for testing. There are five data sets in total: the training datasets ${S}_1$, ${S}_2$, ${S}_3$ for human, yeast, and mouse have 990, 628, and 944 samples, respectively, and the independent testing datasets ${S}_4$, ${S}_5$ for humans and yeasts both have 200 samples. Each dataset has half positive and half negative samples, and there is no crossover between the datasets. Table 1 shows the details of the five datasets.

**Table 1:** The information of training datasets and independent testing datasets.

	Name of datasets	Species	The length of the RNA sequences (bp)	The number of positive samples	The number of the negative samples
Training datasets	${S}_1$	*H. sapiens*	21	495	495
	${S}_2$	*S. cerevisiae*	31	314	314
	${S}_3$	*M. musculus*	21	472	472
Independent testing datasets	${S}_4$	*H. sapiens*	21	100	100
	${S}_5$	*S. cerevisiae*	31	100	100

### Sequence analysis

**Figure 2:**
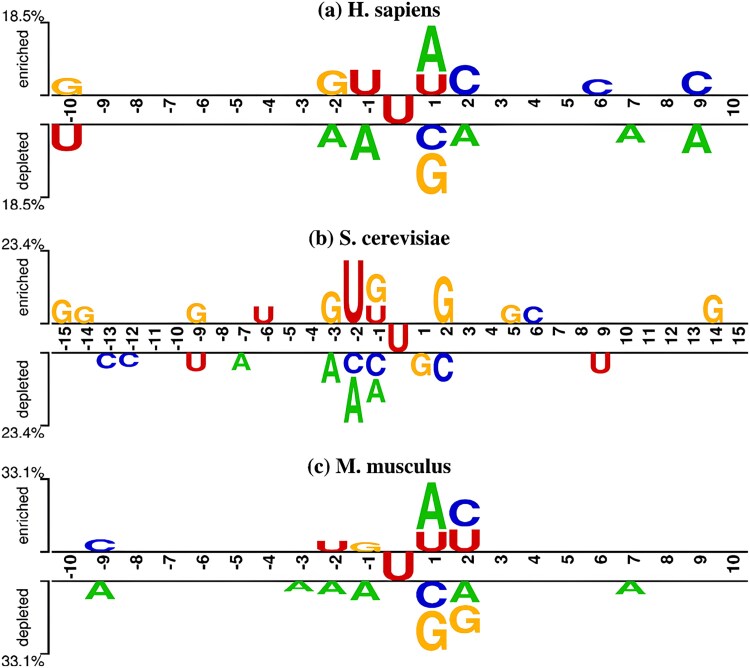
Comparative characterization of pseudouridine and non-pseudouridine sequences in different species.

In order to investigate the appearance pattern of pseudouridine, we utilized two-sample logo tool [22] for sequence analysis. The probability and importance of each nucleotide position in the RNA sequence were calculated using *t*-tests, with an importance threshold set at 0.05 and the center of the sequence (position 0) being the most intermediate nucleotide. The results are shown in Fig. 2. The comparison graph visualizes the enrichment and depletion of specific nucleotides at each position in the sequence, and the height of nucleotide symbols is positively correlated with their frequency of occurrence. The higher the frequency, the higher the symbols; the lower the frequency, the lower the symbols.

The specific patterns are as follows. (i) In the human dataset (*H. sapiens*), there is U enrichment in the middle of the pseudouridine sequence, G enrichment upstream, and C enrichment downstream; multiple As are depleted upstream and downstream of the non-pseudouridine sequence. (ii) In the yeast dataset (*S. cerevisiae*), there are multiple G enrichments upstream and downstream of the pseudouridine sequence and no A enrichments; multiple A and C are depleted upstream of the non-pseudouridine sequence. (iii) In the mouse dataset (*M. musculus*), there is U enrichment upstream and downstream near the middle of the pseudouridine sequence, and multiple A depletions upstream and downstream and G depletions downstream of the non-pseudouridine sequence.

In summary, the pseudouridine sequences of all three species have U enrichment upstream and downstream near the intermediate target U, whereas the negative sample sequences generally have substantial A enrichment.

### Feature extraction method

From the results of sequence analysis, it can be seen that the pseudouridine and non-pseudouridine sequences of different species show specific patterns in nucleotide distribution. For example, in the human, yeast, and mouse datasets, the nucleotide enrichment of pseudouridine sequences and non-pseudouridine sequences at each position differed significantly. The DFT can transform the numerical sequences obtained based on the transformation of nucleotide physicochemical properties from the time domain to the frequency domain, and the obtained frequency domain features can enhance the feature differentiation between pseudouridine sequences and non-pseudouridine sequences. The DFT transformation enables small but critical differences in the sequences to be amplified in the frequency domain, making it easier for the model to capture these differences.

To address the problem of RNA pseudouridine identification, we propose a new feature extraction method in this part. First, for one RNA sequence *S* of length *N*, *S* = (${s}_1$, ${s}_2$, …, ${s}_N$), where ${s}_j\in \Omega$, $\Omega$={A, C, G, U}. We considered two dinucleotide physicochemical properties, free energy and hydrophilicity [9], details of which are shown in Table 2.

**Table 2:** Two types of physicochemical properties of dinucleotides in RNA.

Dinucleotide	Free energy	Hydrophilicity
GG	−3.260	0.170
GA	−2.350	0.100
GC	−3.420	0.260
GU	−2.240	0.270
AG	−2.080	0.080
AA	−0.930	0.040
AC	−2.240	0.140
AU	−1.100	0.140
CG	−2.360	0.350
CA	−2.110	0.210
CC	−3.260	0.490
CU	−2.080	0.520
UG	−2.110	0.340
UA	−1.330	0.210
UC	−2.350	0.480
UU	−0.930	0.440

With Table 2, we can represent each dinucleotide in the RNA sequence using the values of free energy and hydrophilicity, respectively. It will yield two representations, one using free energy and the other using hydrophilicity. Let us take the free energy as an example. First, by the above transformation, the RNA sequence *S* can be transformed into a numerical sequence H, H = (${h}_1$, ${h}_2$, ···,$\kern0.5em {h}_{N-1}$), with the length of the sequence being *N*-1. Then, a DFT is applied to it to convert the numerical sequence into frequency domain features [23]. The formula is as follows:


(1) \begin{equation*} H(k)=\sum \limits_{n=0}^{N-2}h(n){\text{e}}^{-i\left(\frac{2\pi }{N-1}\right) kn},\kern1em k=0,1,2,\cdots, N-2, \end{equation*}

where *H*(*k*) denotes the *k*th value of the transformation (which is in complex form) and *h*(*n*) representing the (*n* + 1)th value in the numerical sequence H (i.e. it is ${h}_{n+1}$).

Eventually, a DFT produces *N*-1 complex numbers *x* + *iy*. Subsequently, the amplitudes of these DFT values are computed using Equation (2).


(2) \begin{equation*} {Z}_i=\sqrt{x_i^2+{y}_i^2},i=1,2,\cdots, N-1. \end{equation*}

In this way, for an RNA sequence, we can get the feature vector Z, where Z = (${\text{Z}}_1$, ${\text{Z}}_2$, ···,$\kern0.5em {\text{Z}}_{N-1}$). Similarly, for hydrophilicity, using the above method, we can also obtain the feature vectors Z and then combine them. Eventually, for an RNA sequence, we can get 2(*N*-1) features. It is worth noting that if the length of each RNA sequence in the dataset is inconsistent, we should take the longest RNA sequence as the standard, and the other sequences are filled with zeros to make the sequence length consistent.

### Convolutional neural network

CNNs were formally introduced by LeCun *et al.* [24] of New York University in 1998. It is a neural network specialized for processing data with grid-like structure, such as time series data (1D grid) and image data (2D pixel grid). CNN extracts features automatically by simulating the visual system of the human brain and adopting a model structure with sequentially alternating convolutional and pooling layers, and it is highly invariant to deformations of the image (e.g. translations, scaling, and skewing).

The basic structure of a CNN consists of an input layer, a convolutional layer, a pooling layer (also called a subsampling or sampling layer), and a fully connected layer. In the convolutional layer, a neuron is connected to only some of the neighboring neurons and the image is feature extracted by a convolutional kernel. The convolution kernel is generally initialized in the form of a matrix of random decimals, which is learned to get reasonable weights during the training process of the network. Neurons of the same feature map share weights, which helps reduce connections between layers of the network, reduce the risk of overfitting, and simplify model complexity. The pooling layer, on the other hand, uses the principle of image local correlation to sample the image between neighborhoods, extracting useful information while reducing the amount of data.

**Figure 3:**
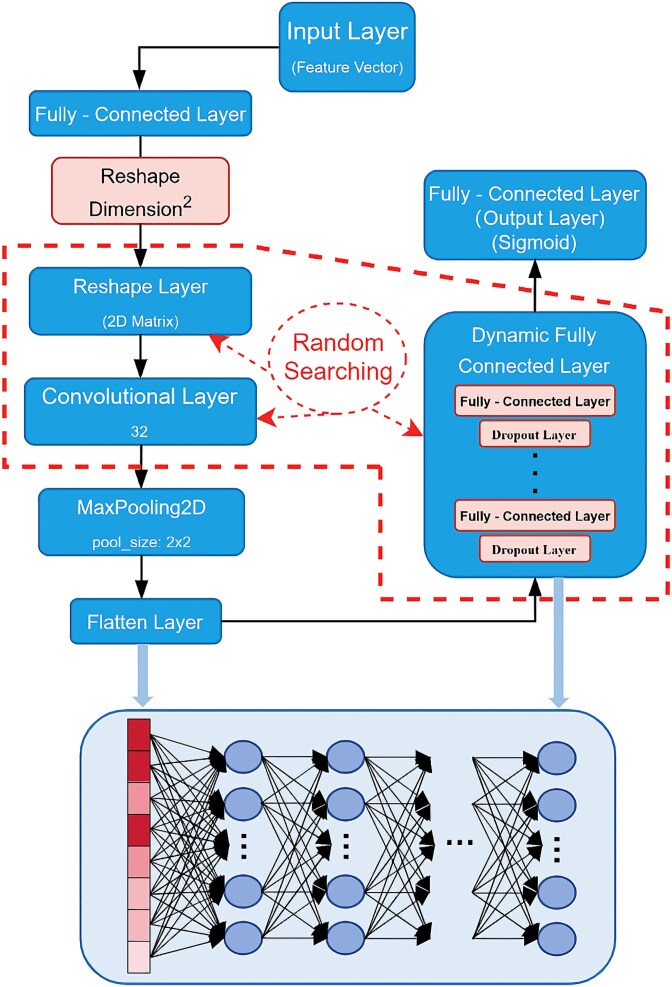
Structure of CNN model.

Figure 3 shows the structure of the CNN model constructed in this paper, which adopts a hybrid convolutional + dynamic fully connected architecture with both global feature integration and local pattern capture capabilities, and uses a dynamic dimensional adaptation mechanism to automatically be compatible with feature inputs of different sizes. The relevant parameters and the number of fully connected layers in the model are optimized by the random search algorithm, and the core structure of the model contains the following key components. (i) The feature engineering layer projects the original 1D features to the high-dimensional space through fully connected mapping, and its dimensionality is controlled by the adjustable parameter reshape_dim, which generates an intermediate feature vector with the shape of (reshape_dim^2^, 1). Subsequently, the vector is converted to a 3D tensor structure of (reshape_dim, reshape_dim, 1) through a 2D reshape operation, providing a standardized input format for subsequent spatial feature extraction. (ii) The feature extraction layer uses a 2D convolutional kernel (conv_kernel) with adjustable parameters for spatial feature learning and captures local feature patterns through a sliding window. The convolution operation is followed by a 2 × 2 maximum pooling layer, which achieves feature map size compression while retaining significant features, effectively reduces computational complexity and enhances translation invariance to form a multiscale feature representation. (iii) The classification decision layer constructs a dynamic fully connected network, whose architecture is flexibly configured by the layer parameter dense_layers and the number of neurons per layer dense_units, and each layer contains 30% dropout to prevent overfitting. The network maps the extracted high-level semantic features to the classification space, realizes decision boundary construction through nonlinear combination, and finally outputs category probability distributions. Parameter tunability also enables the network to adapt to classification tasks of different complexity, balancing model capacity, and overfitting risk.

### Model evaluation

The cross-validation method [25] is a widely used and efficient evaluation strategy. It involves dividing the dataset into K nonoverlapping subsets and performing K independent training and validation cycles. In each cycle, K-1 subsets are used for training, and the remaining subset is used for validation. This approach reduces the chance error from a single data split and provides a more comprehensive and accurate assessment of the model’s generalization ability.

In this experiment, in order to obtain a more superior model, we chose the same assessment method as the developed model to evaluate its performance, i.e. we used the 10-fold cross-validation method. This strategy ensures that our evaluation results are not only accurate and reliable but also helps to make a fair comparison and optimization of the performance of different models.

Meanwhile, in order to assess the performance of the predictor in an all-round way, we used five different evaluation metrics: ACC, sensitivity (Sn), specificity (Sp), Matthew’s correlation coefficient (MCC), and area under the curve (AUC) [26–30]. These metrics reflect the performance characteristics of the predictor from different dimensions, which help us understand the performance of the predictor deeply in various scenarios, thus providing a strong basis for model selection and improvement. The formulas are as follows:


(3) \begin{equation*} \left\{\begin{array}{@{}c} ACC=\frac{TP+ TN}{TP+ TN+ FP+ FN}\\{} Sn=\frac{TP}{TP+ FN}\\{} Sp=\frac{TN}{TN+ FP}\\{} MCC=\frac{TP\times TN- FP\times FN}{\sqrt{\left( TP+ FP\right)\left( TP+ FN\right)\left( TN+ FP\right)\left( TN+ FN\right)}}\end{array}\right., \end{equation*}

where *TP* denotes the number of correctly predicted positive samples, *TN* denotes the number of correctly predicted negative samples, *FN* denotes the number of incorrectly predicted positive samples, and *FP* denotes the number of incorrectly predicted negative samples.

The AUC is a very important evaluation metric in the field of machine learning, which is mainly used to measure the performance of a binary classification model. The value of AUC lies between 0 and 1, and the larger the value, the better the performance of the classifier.

## Experimental results and discussion

### Feature dimensions of each dataset

Using the method proposed in this article for feature extraction, the dimensionality of features extracted from different datasets is shown in Table 3. It can be seen that the feature dimensions of the datasets ${S}_1$, ${S}_3$, and ${S}_4$ are 40 and that the feature dimensions of the datasets ${S}_2$ and ${S}_5$ have a feature dimension of 60.

**Table 3:** Feature dimensions for different datasets.

	Name of datasets	Species	The length of the RNA sequences (bp)	Feature dimension
Training datasets	${S}_1$	*H. sapiens*	21	40
	${S}_2$	*S. cerevisiae*	31	60
	${S}_3$	*M. musculus*	21	40
Independent testing datasets	${S}_4$	*H. sapiens*	21	40
	${S}_5$	*S. cerevisiae*	31	60

### Model training

In order to be able to explore the hyperparameter combinations with higher efficiency and avoid the high computational cost problem that grid search needs to exhaust all combinations, we adopt the random search [31] algorithm for hyperparameter optimization. Among them, the random search algorithm significantly reduces the computational complexity by randomly sampling hyperparameter combinations. In the hyperparameter tuning of complex models such as deep learning (e.g. CNN, RNN), the high efficiency of random search makes it more advantageous, and it can quickly locate better hyperparameter configurations under limited resources to improve model performance.

In this paper, we optimized six types of core parameters to improve the training speed and save time and cost, as follows. (i) Feature reshape dimension (reshape_dim): control the 2D spatial arrangement of the feature matrix, the parameter space is 6 × 6, 7 × 7, and 8 × 8. When the input feature dimension of the original sequence is 60, selecting 8 × 8 = 64 needs to fill four zero values. (ii) Convolution kernel size (conv_kernel): control the size of the sensory field, the parameter space is 3 × 3, 4 × 4, and 5 × 5. (iii) Convolutional kernel step size (conv_stride): affects the feature map downsampling rate, with parameter spaces of 1, 2, and 3. (iv) Layers of dynamic fully connected layers (sense_layers): controls the model complexity, with a parameter space of 1, 2, and 3 layers. (v) Number of units (sense_units) of dynamic fully connected layers: balancing the characterization ability and overfitting risk, with parameter space of 4, 8, 16, and 32 units. (vi) Activation function (activation): ReLU or SeLU.

The search phase was quickly evaluated using five folds of 15 epochs with dual metrics of ACC and MCC, and the final validation was finely trained using 10 folds of 30 epochs.

The optimal parameters of the model were finalized by training on three training sets: human (*H. sapiens*), yeast (*S. cerevisiae*), and mouse (*M. musculus*), as shown in Table 4 below.

**Table 4:** Parameter values of the model on different training sets.

Parameter	*H. sapiens*	*S. cerevisiae*	*M. musculus*
reshape_dim	8 × 8	8 × 8	8 × 8
conv_kernel	5 × 5	4 × 4	4 × 4
conv_stride	3	2	2
sense_layers	2	1	1
sense_units	32	32	32
activation	SeLU	ReLU	ReLU

As can be seen from the table, the values of the reshape_dim and sense_units parameters are the same for all three training sets, and the values of all parameters are the same for the two training sets, *S. cerevisiae* and *M. musculus*. On the *H. sapiens* training set, two fully connected layers were used and the activation was SeLU, while the exception two training sets used only one fully connected layer and the activation was ReLU. It can be seen that the human model is more complex and requires larger convolutional kernels and deeper network hierarchies to improve complex pattern recognition.

### Feature importance analysis

**Figure 4:**
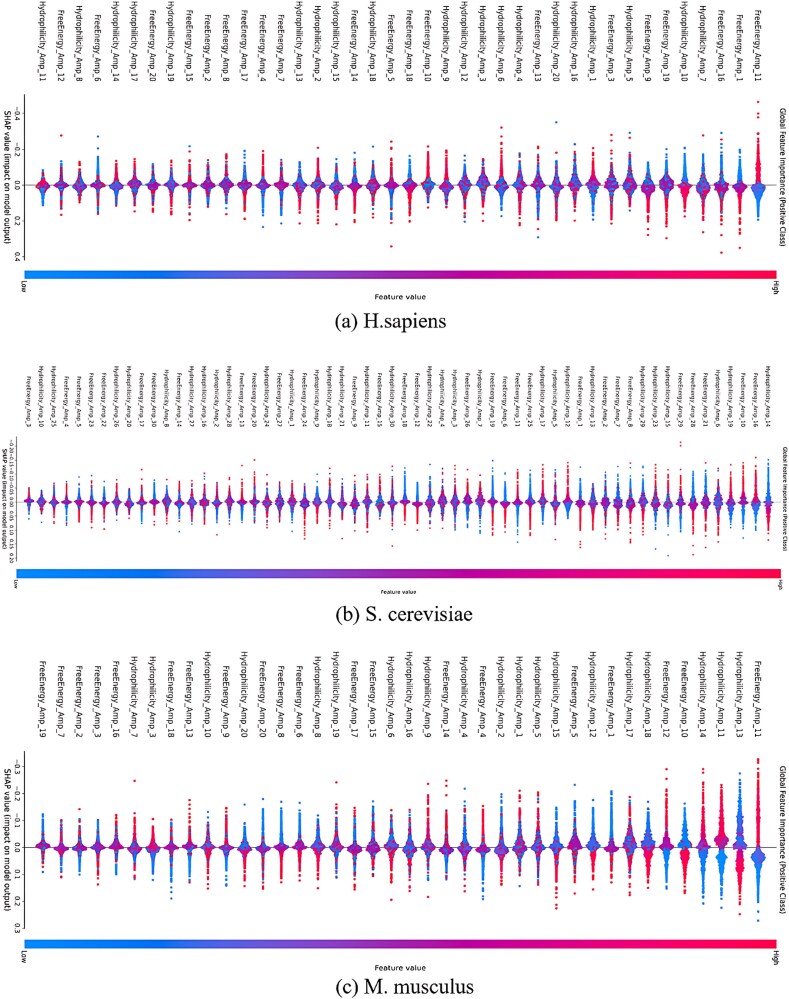
SHAP significance analysis of free energy and hydrophilicity features in different training sets.

For biologists, it is crucial to understand how the model makes decisions and which features have the greatest impact on the prediction results. Therefore, in this paper, we use Shapley additive explanations (SHAP) [32] values to analyze how much each feature contributes to the model’s prediction results to help researchers understand the model’s decision-making process, the results are displayed in Fig. 4.

In Fig. 4, the Y-axis represents the SHAP values, which reflects how much the features affect the model’s output. Positive values indicate that the feature tends to increase the probability of the model predicting a positive class, and negative values indicate that the feature tends to decrease the probability of the model predicting a positive class; the X-axis lists the names of different features; the color of the dots represents the high or low value of the feature, with red denoting a high value of the feature, and blue denoting a low value of the feature. Each feature corresponds to a number of dots, with each dot representing the SHAP value of the feature and the situation of the feature’s value in one sample. The more dispersed the dots are, the greater the influence of the feature on the model output in different samples.

From the figure, it can be seen that in the SHAP analysis of *H. sapiens* training set, the characteristic SHAP values such as “FreeEnergy_Amp_1/11/16” and “Hydrophilicity_Amp_7” are widely distributed with large absolute values, which significantly affect the prediction of pseudouridine (positive class) and dominate the model decision. The SHAP values of features such as “FreeEnergy_Amp_8” and “Hydrophilicity_Amp_11” are concentrated around 0, which have little effect on the prediction of the positive class. On the whole, FreeEnergy_Amp has a higher proportion of important features, while Hydrophilicity_Amp has a more dispersed distribution, and the contribution of the two types of features to the model is different in terms of the way and degree.

On the *S. cerevisiae* training set, the SHAP values of some features (e.g. “FreeEnergy_Amp_16/10/21” and “Hydrophilicity_Amp_14/19/6”) were widely distributed and had large absolute values, indicating that these features had a significant impact on the model’s prediction of the positive class (pseudouridine). The SHAP values of some features (e.g. “FreeEnergy_Amp_5/4/3” and “Hydrophilicity_Amp_25/10”) are highly concentrated around 0, with a very small range of fluctuation, whose effect on the model’s prediction of the positive class is minimal. Meanwhile, the red dots (high eigenvalues) of the “FreeEnergy_Amp” feature are mostly concentrated in the positive SHAP region, while the blue dots (low eigenvalues) are mostly concentrated in the negative SHAP region, which indicates that the level of these eigenvalues is closely related to the direction of the model’s influence on the prediction of the positive category.

On the *M. musculus* training set, FreeEnergy_Amp_11 has the highest SHAP value and contributes the most to the positive class prediction, indicating that the free energy amplitude changes near position 11 of the RNA sequence is critical for pseudouridine identification. Hydrophilicity_Amp_13 is the next most important feature, and hydrophilicity amplitude change at position 13 significantly affects the modeling decision-making. The hydrophilicity feature (Hydrophilicity_Amp_*) occupies three of the top 5 positions, indicating that the local hydrophilicity amplitude of RNA sequences is the core factor for pseudouridine identification. The key features are concentrated in Positions 10–20 (e.g. Positions 11, 13, and 17), which are consistent with the “U-rich upstream and downstream regions” mentioned in the sequence analysis, suggesting that changes in the physicochemical properties of these positions may be the hallmarks of pseudouridine formation.

### Selection of classifiers

**Figure 5:**
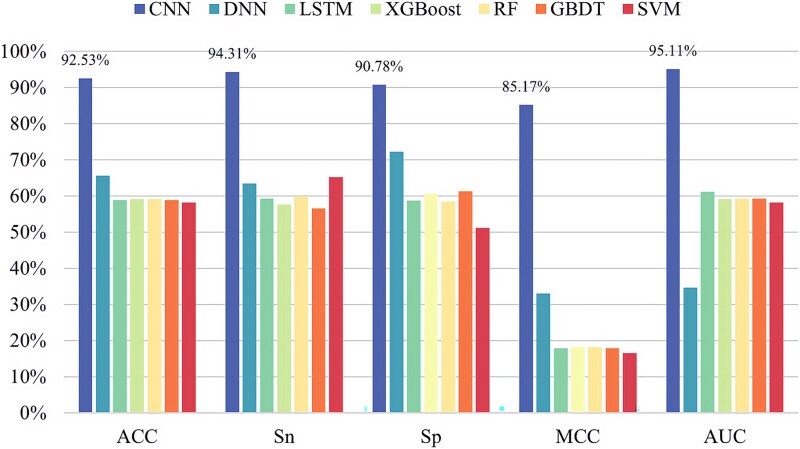
Experimental results of seven classifiers on dataset ${S}_1$.

**Figure 6:**
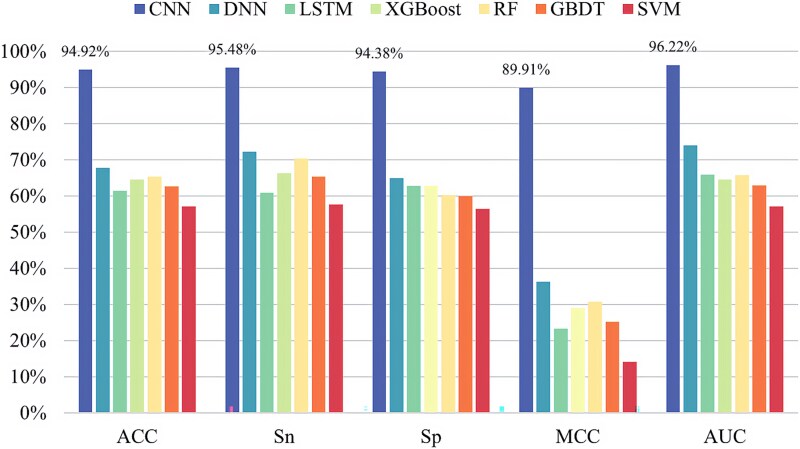
Experimental results of seven classifiers on dataset ${S}_2$.

**Figure 7:**
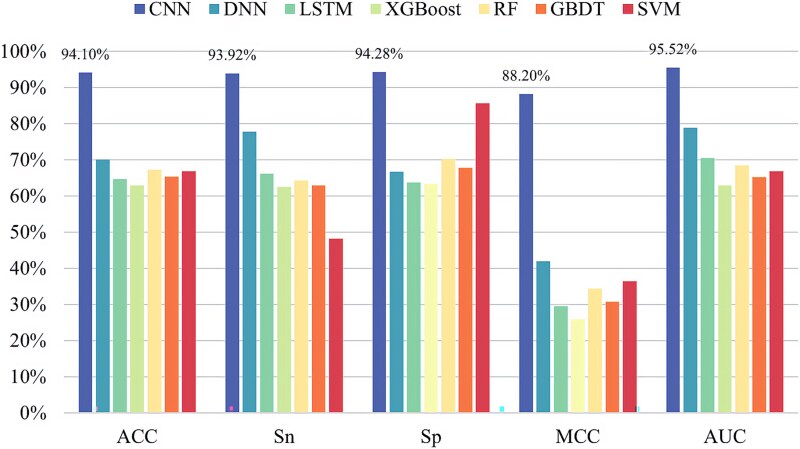
Experimental results of seven classifiers on dataset ${S}_3$.

**Figure 8:**
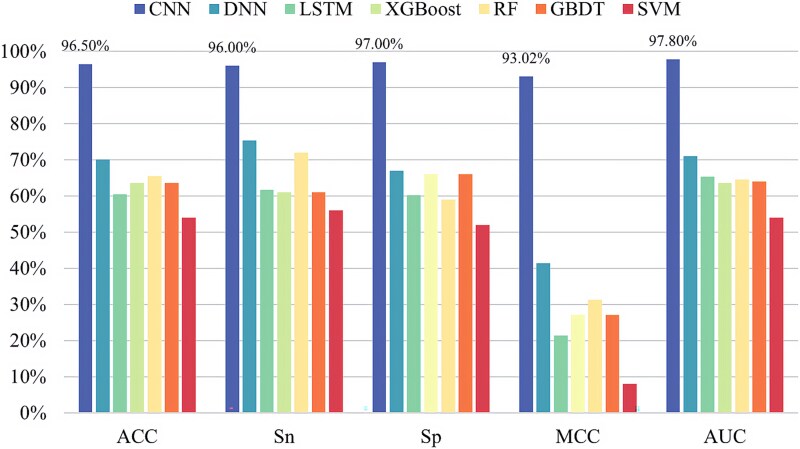
Experimental results of seven classifiers on dataset ${S}_4$.

**Figure 9:**
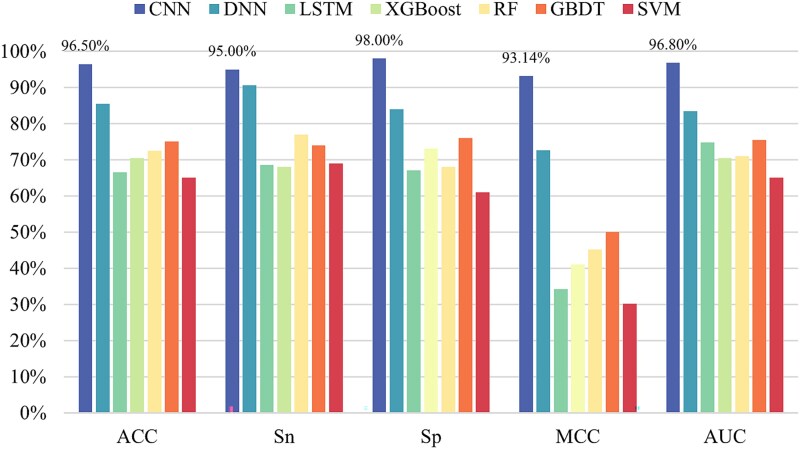
Experimental results of seven classifiers on dataset ${S}_5$.

In order to construct a more effective pseudouridine predictor, seven classifiers are selected for in-depth analysis of pseudouridine data in this paper, namely CNN, deep neural network (DNN) [33], long–short-term memory (LSTM) network [34], XGBoost [35], random forest (RF) [36], gradient-boosted decision tree (GBDT) [37], and SVM [38]. The experimental results are shown in Figs 5–9, demonstrating the specific values of the seven classifiers in terms of ACC, Sn, Sp, MCC, and AUC for the five sets of datasets under 10-fold cross-validation.

From the experimental results, it is evident that the CNN performs best on all the five datasets and the values of ACC, Sn, Sp, MCC, and AUC are much greater than the values of other classifiers. The above experiments demonstrate the superiority of CNNs in RNA pseudouridine identification.

### Comparison with previous models

Although past studies have constructed many identification models for RNA pseudouridine, there is still some optimization space for these models. Based on these existing prediction models, we propose a new identification method. In order to fully demonstrate the usefulness of our model, it is crucial to compare it with previous methods.

In Tables 5 and 6, we detail the evaluation metrics values of the predictors used to predict the RNA pseudouridine models on both the training set and the independent test set. These models include iPseU-CNN [19], XG-PseU [11], RF-PseU [12], iPseU-TWSVM [13], PA-PseU [14], and the new model proposed in this paper, RSCNN-PseU. Among them, the iPseU-CNN model is also based on CNN to recognize RNA pseudouridine, but its evaluation index value is based on the one obtained by the 5-fold cross-validation (5-CV) method. Therefore, in order to make a fairer comparison, we also performed a 5-CV.

**Table 5:** Comparison of the results of the training set of each model.

Species	Classifier	Training datasets				
		ACC	Sn	Sp	MCC	AUC
*H. sapiens* (${S}_1$)	iPseU-CNN(5-CV)	0.667	0.65	0.688	0.34	N/A
	XG-PseU(10-CV)	0.661	0.635	0.687	0.32	0.7
	RF-PseU(10-CV)	0.643	0.661	0.626	0.29	0.7
	iPseU-TWSVM(10-CV)	0.65	0.697	0.602	0.301	0.682
	PA-PseU(10-CV)	0.854	N/A	N/A	N/A	N/A
	RSCNN-PseU(10-CV)	**0.9253**	**0.9431**	**0.9078**	**0.8517**	**0.9511**
	RSCNN-PseU(5-CV)	0.8616	0.8713	0.8548	0.7247	0.8966
*S. cerevisiae* (${S}_2$)	iPseU-CNN(5-CV)	0.682	0.664	0.705	0.37	N/A
	XG-PseU(10-CV)	0.682	0.668	0.695	0.37	0.77
	RF-PseU(10-CV)	0.748	0.772	0.724	0.49	0.81
	iPseU-TWSVM(10-CV)	0.722	0.656	0.786	0.45	0.758
	PA-PseU(10-CV)	0.939	N/A	N/A	N/A	N/A
	RSCNN-PseU(10-CV)	**0.9492**	**0.9548**	**0.9438**	**0.8991**	**0.9622**
	RSCNN-PseU(5-CV)	0.9032	0.9026	0.9038	0.8064	0.9147
*M. musculus* (${S}_3$)	iPseU-CNN(5-CV)	0.718	0.748	0.691	0.44	N/A
	XG-PseU(10-CV)	0.72	0.765	0.676	0.45	0.74
	RF-PseU(10-CV)	0.748	0.731	0.765	0.5	0.796
	iPseU-TWSVM(10-CV)	0.728	0.795	0.661	0.462	0.775
	PA-PseU(10-CV)	0.845	N/A	N/A	N/A	N/A
	RSCNN-PseU(10-CV)	**0.941**	**0.9392**	**0.9428**	**0.882**	**0.9552**
	RSCNN-PseU(5-CV)	0.8942	0.8967	0.892	0.7803	0.9209

*Note:* Bold values indicate the highest score for each performance metric within the same species. iPseU-CNN relies on 5-CV, which may underestimate generalization performance compared to 10-CV. PA-PseU lacks reported Sn and Sp, limiting direct comparison. XG-PseU shows lower AUC (0.7–0.77) due to insufficient feature representation. iPseU-TWSVM model uses seven different feature extraction methods (Kmer, PC-PseDNC-General, ANF, EIIP, ENAC, nucleotide chemical property, and NBP), which results in a high number of extracted feature dimensions and high model complexity. In contrast, the RSCNN-PseU model only needs to extract a small number of features to obtain a high ACC.

**Table 6:** Comparison of the results of independent test set of each model.

Species	Classifier	Independent testing datasets				
		ACC	Sn	Sp	MCC	AUC
*H. sapiens* (${S}_4$)	iPseU-CNN(5-CV)	0.69	0.777	0.68	0.4	N/A
	XG-PseU(10-CV)	0.675	N/A	0.608	N/A	N/A
	RF-PseU(10-CV)	0.75	0.78	0.72	0.5	0.8
	iPseU-TWSVM(10-CV)	0.763	0.825	0.7	0.529	0.786
	PA-PseU(10-CV)	0.81	N/A	N/A	N/A	N/A
	RSCNN-PseU(10-CV)	**0.965**	**0.96**	**0.97**	**0.9302**	**0.978**
	RSCNN-PseU(5-CV)	0.915	0.92	0.91	0.8304	0.931
*S. cerevisiae* (${S}_5$)	iPseU-CNN(5-CV)	0.735	0.688	0.778	0.47	N/A
	XG-PseU(10-CV)	0.71	N/A	N/A	N/A	N/A
	RF-PseU(10-CV)	0.77	0.75	0.79	0.54	0.838
	iPseU-TWSVM(10-CV)	0.825	0.85	0.8	0.65	0.905
	PA-PseU(10-CV)	0.81	N/A	N/A	N/A	N/A
	RSCNN-PseU(10-CV)	**0.965**	**0.95**	**0.98**	**0.9314**	**0.968**
	RSCNN-PseU(5-CV)	0.915	0.90	0.93	0.8303	0.9195

*Note*: Bold values indicate the highest score for each performance metric within the same species. The values of the evaluation metrics in the independent test set are predicted by the model trained in the training set.

From Tables 5 and 6, we can see that our model RSCNN-PseU has the highest values on all five evaluation metrics. Among them, in the training set (${S}_1,\kern0.5em {S}_2$, and ${S}_3$), our model is 0.9253, 0.9492, and 0.941 in terms of ACC, which is 0.0713, 0.0102, and 0.096 higher than PA-PseU, respectively. While under the 5-CV, RSCNN-PseU is 0.1946, 0.2212 and 0.1762 higher than iPseU-CNN in terms of ACC, respectively. In the independent test set (${S}_4$ and${S}_5$), RSCNN-PseU has ACCs of both 0.965, higher in ACC than the model PA-PseU by 0.155, and iPseU-TWSVM by 0.202 and 0.14, respectively. While under 5-CV, RSCNN-PseU outperformed iPseU-CNN by 0.225 and 0.18 in ACC, respectively.

The above tables also show that the MCC values (0.8517–0.9314) of RSCNN-PseU indicate a strong correlation between the predicted labels and the real labels, which is significantly better than that of iPseU-TWSVM (MCC: 0.301–0.65). Moreover, the AUC values (0.9511–0.978) also demonstrate the robustness of our feature extraction method. For example, on dataset ${S}_4$, the AUC value of RSCNN-PseU is 0.978, while RF-PseU and iPseU-TWSVM are only 0.8 and 0.786, respectively, which suggests that RSCNN-PseU is effective in capturing sequential patterns that are crucial for pseudouridine identification.

Table 7 lists the average ACCs of different models for identifying the three species on the training set and independent test set, which can reflect the cross-species prediction ability of the models. From the table, it can be seen that RSCNN-PseU has an average ACC of 0.9385 and 0.965 on the training set and independent test set, respectively, which are 0.0652 and 0.155 higher than PA-PseU. Under 5-CV, our model achieved average ACCs of 0.8863 and 0.915 on the training and independent test sets, respectively, which are 0.1973 and 0.202 higher than iPseU-CNN. And the difference between RSCNN-PseU for 5-CV and 10-fold cross-validation is also very small, indicating that the model has strong generalization ability. The above results also fully prove that the newly proposed model is more accurate and effective in identifying RNA pseudouridine.

**Table 7:** Comparison of average ACC between RSCNN-PseU and other models on training and independent test sets.

	Training datasets	Independent testing datasets
RSCNN-PseU(5-CV)	0.8863	0.915
RSCNN-PseU(10-CV)	**0.9385**	**0.965**
PA-PseU(10-CV)	0.8733	0.81
iPseU-TWSVM(10-CV)	0.7	0.794
RF-PseU(10-CV)	0.713	0.76
XG-PseU(10-CV)	0.687	0.693
iPseU-CNN(5-CV)	0.689	0.713

*Note*: Bold values indicate the highest accuracy (ACC) scores across all models in their respective dataset columns.

## Conclusion

In this paper, we propose a new model RSCNN-PseU for pseudouridine identification. First, the original sequence is transformed into a numerical sequence based on the two physicochemical properties of dinucleotides, free energy and hydrophilicity; then, it is subjected to a DFT and the amplitude of each DFT value is calculated. In this way, for an RNA sequence of length N, we can obtain 2(*N*-1) features. We also performed a feature importance analysis, visualized by SHAP analysis to verify the validity of these features chosen to construct the model in the paper. Finally, comparison experiments with other classifiers including DNN, LSTM, XGBoost, RF, GBDT, and SVM were conducted and finally CNN was chosen for model prediction, and the random search algorithm is used to dynamically determine the number of fully connected layers and optimize the hyperparameters, which achieves adaptive regulation of model complexity and adapts to the needs of different species and datasets. Compared with previous models, the results not only show that the new feature extraction method is effective but also prove the superiority of our model RSCNN-PseU in RNA pseudouridine identification.

While RSCNN-PseU has shown high performance in experiments, it has certain limitations. Firstly, the feature extraction requires fixed-length RNA sequences, which may lose information during zero-padding for shorter sequences. Secondly, we only considered two dinucleotide properties (free energy and hydrophilicity), while higher-order physicochemical interactions (e.g. trinucleotide effects) could further improve ACC. Finally, the model architecture lacks integration with metaheuristic optimization algorithms (e.g. PSO or genetic algorithms) that could improve generalization across diverse biological contexts. Future iterations will address these constraints through three strategic enhancements: (i) implementing dynamic sequence length adaptation mechanisms to eliminate padding artifacts, (ii) expanding feature engineering to capture RNA modification-related patterns at multiple nucleobase interaction scales, and (iii) embedding hybrid optimization modules to automatically tune hyperparameters and feature weights.

**Key Points**

- Novel feature extraction: uses dinucleotide physicochemical properties (free energy and hydrophilicity) transformed via discrete Fourier transform (DFT) to generate 2(*N*-1) numerical features from RNA sequences.
- Feature validation: employs Shapley additive explanation analysis to demonstrate the importance and validity of the chosen physicochemical features for pseudouridine identification.
- Optimized convolutional neural network (CNN) architecture: selects CNN as the core predictor after comparative evaluation against deep neural network, long–short-term memory network, XGBoost, random forest, gradient-boosted decision tree, and support vector machine and uses random search to dynamically optimize hyperparameters and the number of fully connected layers.
- Superior performance: achieves enhanced performance in RNA pseudouridine identification compared to previous models, demonstrating both the effectiveness of the new feature extraction method and the overall superiority of the RSCNN-PseU model.

## Data Availability

Relevant data and model code are available from the website (https://github.com/ZERO-JJ/RSCNN-PseU-).
